# Significance of *CYCLOOXYGENASE*-*2(COX*-*2), PERIOSTIN (POSTN)* and *INTERLEUKIN*-*4(IL*-*4)* gene expression in the pathogenesis of chronic rhinosinusitis with nasal polyps

**DOI:** 10.1007/s00405-014-3481-9

**Published:** 2015-01-09

**Authors:** Jarosław Miłoński, Hanna Zielińska-Bliźniewska, Karolina Przybyłowska, Piotr Pietkiewicz, Barbara Korzycka-Zaborowska, Ireneusz Majsterek, Jurek Olszewski

**Affiliations:** 1 Department of Otolaryngology, Laryngological Oncology, Audiology and Phoniatrics, Medical University of Lodz, Zeromskiego 113, 90-549 Lodz, Poland; 2 Department of Chemistry and Clinical Biochemistry, Medical University of Lodz, Lodz, Poland

**Keywords:** Gene expression, Chronic rhinosinusitis with nasal polyps

## Abstract

The purpose of this paper was to evaluate the level of *Cyclooxygenase*-*2 (COX*-*2), Periostin (POSTN)* and *Interleukin*-*4(IL*-*4)* gene expression in patients with chronic rhinosinusitis with nasal polyps, without polyps and with a nasal septum deviation. The tests were performed on 63 patients (24 women and 39 men) with chronic rhinosinusitis and polyps (CRSwP—study group I), with determination of the *COX*-*2, POSTN* and *IL*-*4* gene expression; an allergy was diagnosed in 38 cases. The reference groups were patients with chronic rhinosinusitis without polyps––CRS (*n* = 23, including 14 women and 9 men) and patients with nasal septum deviation––DSN (*n* = 18, including 9 women and 9 men). The expression level was determined in the polyp tissue and the mucosa of paranasal sinus collected during an FESS. The expression level of studied genes was also evaluated in the material. Immediately after being collected, the tissue fragments were placed in test tubes with 1 ml of RNAlater (*Qiagen,* Hilden, *Germany*) preventing the degradation of RNA and frozen at −70 °C. The studies revealed an increased level of *POSTN, IL*-*4* gene expression and a decreased level of *COX*-*2 gene* expression that may be associated with the development of chronic rhinosinusitis with nasal polyps. An analysis of the expression level indicates the participation of *POSTN* and *IL*-*4* in the development of chronic rhinosinusitis with nasal polyps in patients with atopy.

## Introduction

For a very long time, nasal polyps were considered to be adenomas, and their development was associated with desmoplasia. There are numerous hypotheses on the formation of polyps [1–5]. In 1896, Hayek assumed that polyps formed as a consequence of an exudate which pushes the mucosa out, leading to local congestion, oedema and blood retention in the vessels. In 1907, Yonge described another reason, cystic distension of the mucosal glands (caused by their excessive activity and ongoing chronic inflammation) which, supposedly, pressed mechanically against local blood vessels and the glands themselves, leading to the oedema and congestion.

More recent hypotheses emphasise the significance of isolated damage to the epithelium and its basement membrane, related to the inflammation process as a sign to start polypogenesis, e.g., a 1991 hypothesis by Larsen and Tos [6].

In 1997, Bernstein [7] presented an interesting concept of disorders in the function of ion channels in the epithelial cellular membrane, including excessive absorption of Na^+^ and increased transmission of CL^−^. He claimed that by increasing the transport of water into interstitial spaces, the increased transepithelial ion transport induced oedema and the formation of nasal polyps.

Currently, the following factors are listed as pathogenic for chronic rhinosinusitis with/without nasal polyps:

- exogenous factors: viruses, bacteria, fungi, drugs, injuries, toxic substances, or environmental pollution;
- general endogenous factors: allergy, oversensitivity to acetylsalicylic acid and its derivatives, hormonal disorders, laryngopharyngeal reflux, diseases with the formation of granulation tissue, immunity disorders, genetic syndromes of ciliary disorders (Kartagener syndrome, cystic fibrosis), or oedemal causes;
- local endogenous factors: anatomic abnormalities (enlarged ethmoid bulla, aerated and distended middle nasal concha, and nasal septum deviation), tumours, or acquired syndromes of ciliary disorders in the respiratory epithelium [8–11].

Polyps, as part of the course of chronic rhinosinusitis, are considered primarily to be the consequences of advanced inflammatory processes [12–15]. However, the actual processes responsible for their development remain unclear.

Current studies focus on explaining the role of mucosal epithelium in the nose and sinuses; its lesions (similarly as in asthma) may start and then sustain the pathophysiological processes [14]. The significance of the epithelium in the development of respiratory diseases results from its immunoregulative function and biological activity, as well as the possibility of releasing numerous mediators. Currently, it is assumed that the development of nasal polyps consists of:

- disorder of the epithelial cell function,
- eosinophilic inflammation of the mucosa.

The purpose of this paper was to evaluate the level of *CYCLOOXYGENASE*-*2 (COX*-*2), PERIOSTIN (POSTN), INTERLEUKIN*-*4 (IL*-*4)* gene expression in patients with chronic rhinosinusitis: with nasal polyps, without polyps and with nasal septum deviation.

## Materials and methods

Out of 300 patients (140 women and 160 men) hospitalised in the Department of Otolaryngology and Laryngological Oncology, Medical University of Lodz in 2010–2012 qualified for surgical endoscopic treatment of the nose and paranasal sinuses, a total of 63 patients with chronic rhinosinusitis with polyps (CRS with P) were randomly selected for study group I, for which the level of *COX*-*2* and *POSTN* gene expression was to be determined. This group included 24 women and 39 men (mean age 48.7 ± 13.3 years) with a diagnosed allergy in 38 cases (60 %). The reference groups were: patients with CRS without P––CRS (n = 23, including 14 women and 9 men, mean age 46.4 ± 13.2 years) and patients with nasal septum deviation—DSN (*n* = 18, including 9 women and 9 men, mean age 42.8 ± 13.9 years—Table 1).

**Table 1:** Characteristics of study group (gender)

Gender	Groups						Summary
	I Study (CRSwP)		II Control 1 (CRS)		II Control 2 (DNS)		
	*n*	%	*n*	%	*n*	%	
Man	39	61.9	9	39.1	9	50.0	57
Woman	24	38.1	14	60.9	9	50.0	47
Summary	63	100.0	23	100.0	18	100.0	104

The expression level was determined in the polyp tissue and the mucosa of paranasal sinus collected during an endoscopic FESS. Fragments of the mucosa from paranasal sinuses with CRS without Ps and a fragment of the mucosa from the lower nasal concha in patients with nasal septum deviation were collected during the planned endoscopic procedures. The expression level of the genes was also evaluated in the material.

Immediately after being collected, the tissue fragments were placed in test tubes with 1 ml of RNAlater (Qiagen, Hilden, Germany) to prevent the degradation of RNA, and frozen at −70 °C. The studies were approved by the Bioethics Commission at the Medical University of Lodz (Decision no. RNN/40/09/KB of 6th January 2009), and consent of each patient was obtained.

### Isolation of RNA

The total RNA from the collected tissue fragments was isolated with TriPure Isolation Reagent (Roche Diagnostics GmbH, Mannheim, Germany) according to the manufacturer’s recommendations.

### Qualitative and quantitative assessment of mRNA

The concentration of RNA and clarity of obtained samples were assessed spectrophotometrically. The obtained RNA samples were diluted 50 times in H_2_O-DEPC. The reading was performed with three wavelengths: 260 nm—maximum absorption for RNA and 280 nm—maximum absorption for proteins against the reference solution (in this case, 100 μl of DEPC water).

### Reverse transcription reaction

The synthesis of cDNA on the matrix of isolated RNA was performed with the use of a commercial RevertAid First Strand cDNA Synthesis Kit (Thermo Scientific, Waltham, MA, USA) with the use of reverse transcriptase *M*-*MuLV* (Moloney murine leukaemia virus).

The results of a gel densitometry were used to specify the relative amount of mRNA of the *COX*-*2, POSTN* and *IL*-*4 genes*. The level of analysed genes was standardised against the reference gene, *GAPDH*.

### Statistic methods

The collected data were compiled using descriptive methods and statistical conclusion methods.

Spearman’s rank correlation coefficient was used to examine whether there is a relationship between the studied features and the level of gene expression.

The differences between mean values (or frequencies) and the relations between characteristics for which the calculated value of the test was equal or greater than the critical value, based on appropriate tables, were considered statistically significant, with the correct number of degrees of freedom and an error probability of *p* < 0.05.

## Results

Comparing the level of *COX*-*2* expression between the tissues from those patients with chronic rhinosinusitis, with and without polyps, with the expression level in tissues from the control group (DSN), a significantly higher level in the tissues with chronic rhinosinusitis was shown. It has been observed that the *COX*-*2* expression level was higher in a statistically significant manner in the mucosa of patients with CRS without Ps as compared to the expression level in the mucosa and polyp tissue in patients with CRS with P. Moreover, in the polyp tissue the *COX*-*2* expression level was significantly lower than in the mucosa with CRS with P (Table 2). No relationship between the differences in the *COX*-*2* expression level and presence of atopy in patients with CRS and P was shown, neither in the polyp tissue nor in the mucosa (Table 3).

**Table 2:** The level of *COX*-*2* gene expression in patients with chronic rhinosinusitis with nasal polyps (CRSwP), without nasal polyps (CRS) and in patients with nasal septum deviation (DNS)

CRSwP (*n* = 63)		CRS (*n* = 23) mucosa mediana (25 %; 75 %)	DNS (*n* = 18) mucosa mediana (25 %; 75 %)
Polyp mediana (25 %; 75 %)	Mucosa mediana (25 %; 75 %)		
0.697 (0.561; 0.816)	0.770 (0.727; 0.897) vs polyp ***p*** **<** **0.007**	1.236 (1.022; 1.386) vs polyp CRSw P ***p*** **<** **0.001** vs mucosa CRSwP ***p*** **<** **0.001**	0.400 (0.315, 0.470) vs polyp CRSwP ***p*** **<** **0.001** vs mucosa CRSwP ***P*** **<** **0.001** vs mucosa CRS ***p*** **<** **0.001**

**Table 3:** The level of *COX*-*2* gene expression in patients with ChRS with Ps, considering the presence of atopy

CRSwP	CRSwP with atopy (*n* = 38)	CRSwP without atopy (*n* = 25)	*P*
Polyp Mucosa	0.697 (0.548; 0.786) 0.767 (0.724; 0.808)	0.697 (0.540; 0.837) 0.797 (0.729; 1.268)	0.354 0.124

The analysis of *POSTN* gene expression indicated a significantly higher level of mRNA in polyps and nasal mucosa of those patients with CRS with Ps (CRSwP) and patients with CRS without Ps (CRS), as compared to the level in the nasal mucosa from the control group (DSN). Moreover, it was observed that the POSTN mRNA level was significantly higher in polyps compared to the mucosa of patients with CRS with Ps (CRSwP) and without Ps (CRS), whereas no differences in the *POSTN* expression were observed in comparisons of the mucosa of these two groups of patients (Table 4). The high expression of the *POSTN* gene in patients with ChRS with Ps was related to the presence of an allergy (Table 5).

**Table 4:** The level of *POSTN* gene expression in patients with chronic rhinosinusitis with nasal polyps (CRSwP), without nasal polyps (CRS) and in patients with nasal septum deviation (DNS)

CRSwP (*n* = 63)		CRS (*n* = 23) mucosa mediana (25 %; 75 %)	DNS (*n* = 18) mucosa mediana (25 %; 75 %)
Polyp mediana (25 %; 75 %)	Mucosa mediana (25 %; 75 %)		
2.480 (2.395; 2.743)	0.520 (0.460; 0.590) vs polyp *p* < 0.001	0.524 (0.454; 0.626) vs polyp CRSwP *p* < 0.001 vs mucosa CRSwP *p* = 0.178	0.425 (0.355; 0.503) vs polyp CRSwP *p* < 0.001 vs mucosa CRSwP *P* = 0.019 vs mucosa CRS *p* = 0.031

**Table 5:** The level of *POSTN* gene expression in patients with ChRS with Ps, considering the presence of atopy

*POSTN* mediana (25 %; 75 %)	CRSwP with atopy (*n* = 38)	CRSwP without atopy (*n* = 25)	*P*
Polyp	2.590 (2.450; 3.000)	2.420 (2.190; 2.550)	**<0.001**
Mucosa	0.520 (0.430; 0.590)	0.525 (0.470; 0.620)	**0.456**

The analysis of *IL*-*4* gene expression indicated a significantly higher level of this cytokine in the polyp tissue and nasal mucosa of the patients with CRS with Ps (CRSwP), as compared to the level in the nasal mucosa from those patients with CRS without Ps (CRS) and from the control group (DSN). No statistically significant differences in the level of *IL*-*4* between the polyp tissue and mucosa of those patients with CRS with Ps (CRSwP) were found. A statistically significant increase of expression in the nasal mucosa of those patients with CRS without Ps (CRS) as compared to the control group was observed (Table 6). The high expression of *IL*-*4* gene in patients with CRS with Ps (CRSwP) was related to the presence of an allergy (Table 7).

**Table 6:** The level of *IL*-*4* gene expression in patients with chronic rhinosinusitis with nasal polyps (CRSwP), without nasal polyps (CRS) and in patients with nasal septum deviation (DNS)

CRSwP (*n* = 63)		CRS (*n* = 23) mucosa mediana (25 %; 75 %)	DNS (*n* = 18) mucosa mediana (25 %; 75 %)
Polyp mediana (25 %; 75 %)	Mucosa mediana (25 %; 75 %)		
0.821 (0.747; 1.117)	0.818 (0.732; 1.039) vs polyp *p* = 0.189	0.747 (0.529; 0.977) vs polyp **CRSwP p < 0.001** vs mucosa **CRSwP p = 0.014**	0.116 (0.106; 0.161) vs polyp **CRSwP** **p < 0.001** vs mucosa **CRSwP** **P < 0.001** vs mucosa CRS **p < 0.001**

**Table 7:** The level of *IL*-*4* gene expression in patients with ChRS with Ps, considering the presence of atopy

CRSwP	CRSwP with atopy (*n* = 38)	CRSwP without atopy (*n* = 25)	*P*
Polyp Mucosa	1.056 (0.797; 1.272) 0.916 (0.816; 1.119)	0.777 (0.719; 0.816) 0.736 (0.679; 0.760)	**<0.001** **<0.001**

## Discussion

The *COX*-*2* gene is one of the best researched genes with regard to expression and related mechanisms. Based on numerous experimental studies, it has been proven that an increase in *COX*-*2* expression is observed in many diseases and types of cancer in people. The disorders in the regulation of cyclooxygenase-1 (*COX*-*1*) and cyclooxygenase-2 (*COX*-*2*) have been described by numerous researchers in the nasal polyps of patients with AIA (*Aspirin*-*Induced Asthma*). However, it is unclear whether these disorders are characteristic only of nasal polyps or the entire mucosa of the upper respiratory tract in patients with the aspirin triad (nasal polyps + intolerance of salicylates + asthma). The studies undertaken so far have proved ambiguous, and many indicate an increased COX expression in the tissues of nasal polyps [16–20].

Recent studies by Roca-Ferrer et al. [21] present the differences in *COX*-*2* expression between the polyp tissue and control mucosa. Using three methods of expression analysis (ELISA, Western blot and immunofluorescence), no significant increase of *COX*-*2* expression was observed in those patients with Aspirin-Induced Asthma (AIA). It was observed that IL-1β significantly stimulates the production of PGE2 prostaglandin in fibroblasts from the control of mucosa, but there was no significant effect on fibroblasts obtained from the mucosa of patients with AIA. The stimulation of IL-1β activated the increased expression of *COX*-*1* and *COX*-*2* in fibroblasts obtained from the control (of nasal mucosa from patients without AIA), but not in fibroblasts from the mucosa of patients with AIA. Thus, these data suggest that the disorders of the (COX-1) and (COX-2) cyclooxygenase path apply to the entire mucosa of patients with the aspirin triad, instead of being associated only with nasal polyps and the mucosa of the upper respiratory tract in patients diagnosed with the aspirin triad, and they cannot be associated with the healthy mucosa of patients without the triad [17].

In the authors’ own studies, comparing the level of *COX*-*2* expression between the tissues from the patients with ChRS, with and without nasal polyps, with the expression level in tissues from the control group (DSN), a significantly higher level in the tissues with chronic rhinosinusitis was shown. It has been observed that the *COX*-*2* expression level was higher in a statistically significant manner in the mucosa of patients with ChRS without Ps as compared to the expression level in the mucosa and polyp tissue in patients with ChRS with Ps. Moreover, in the polyp tissue, the *COX*-*2* expression level was significantly lower than in the mucosa with ChRS with Ps. A relationship between the decreased *COX*-*2* expression level and oversensitivity to ASA and NSAIDs in the polyp tissue and mucosa with ChRS with Ps was observed.

The next studied gene is PERIOSTIN (POSTN), whose excessive expression has recently been shown in other types of tissues, including cerebral tissue, sperm or T lymphocytes, and myocardium, taking part in the remodelling following a myocardial infarction.

Takayama et al. [22] showed that *PERIOSTIN* secreted by pulmonary fibroblasts in response to *IL*-*4* and *IL*-*13* is a new component of the subepithelial fibrosis in asthma. Many authors [23, 24] believe that chronic rhinosinusitis with nasal polyps shares a pathogenetic background with asthma, while subepithelial fibrosis plays an important role in both.

Jia et al. [25] also confirmed the role of *periostin* as a biomarker in chronic eosinophilic respiratory track inflammation in patients with asthma.

Norris et al. [26] showed that *POSTN* is expressed in normal mucosa. However, its overexpression may be a significant factor contributing to the pathogenesis of nasal polyps [27]. Moreover, the results of tests with the use of micromatrix comparing the gene expression levels in the tissue of nasal polyps from patients with chronic rhinosinusitis and bronchial asthma showed a characteristic increase in the POSTN gene expression for the studied group of patients, differing from the control group.

Ishida et al. [28] confirmed the enhanced expression of PERIOSTIN in the mucosal tissues of patients with allergic rhinitis, and in the mucosal tissues from the sinuses of patients with CRS and nasal polyps, as compared to the control group. Moreover, the POSTN expression was significantly higher in those patients with CRS and nasal polyps than in those patients with allergic rhinitis. Studies by Ishida et al. suggest that PERIOSTIN plays an important role in the remodelling of mucosa in allergic rhinitis and the formation of the polyp in the course of chronic rhinosinusitis and aspirin-induced asthma (AIA) [28, 29].

The results of the authors’ own studies indicated a significantly higher level of *POSTN* expression in polyps and nasal mucosa of those patients with CRS with Ps (study group) and those patients with CRS without Ps (control group I), as compared to the level in the nasal mucosa from control group II (DSN). Moreover, it was observed that the *POSTN* mRNA level was significantly higher in polyps as compared to the mucosa of patients with CRS with/without Ps, whereas no differences in the *POSTN* expression were observed while comparing the mucosa of these two groups of patients. The high expression of the *POSTN* gene in patients with ChRS with Ps was related to the presence of atopy as observed in the polyp tissues, whereas no similar dependence was found in the mucosa of these patients.

They were studied to assess the significance of fibroblasts in the formation of polyps in the course of chronic rhinosinusitis and the role of *IL*-*4* in the activation of fibroblasts. It was observed that *IL*-*4* influences the regulation of leukotriene receptor expression, and the proliferation of fibroblasts and their secretion of proinflammatory cytokines participating in the formation of polyps.

## Conclusions

1. An increased level of *POSTN, IL*-*4* gene expression and a decreased level of COX-2 gene expression may be related to the development of chronic rhinosinusitis with nasal polyps.
2. An analysis of the expression level indicates the participation of *POSTN* and *IL*-*4* in the development of chronic rhinosinusitis with nasal polyps in patients with atopy.
